# Advances in quantitative methods and modeling for complex generic drugs and opportunities to hybridize learnings between innovator and generic drug developers

**DOI:** 10.1002/psp4.12968

**Published:** 2023-04-27

**Authors:** Priya Jayachandran

**Affiliations:** ^1^ Clinical Pharmacology & Bioanalytics Pfizer Inc. Cambridge MA USA

Approximately 90% of prescriptions dispensed in the United States are generic drugs.^1^ The continued availability of safe, effective, and high‐quality generics is a top priority of the US Food and Drug Administration (FDA). As the Generic Drug User Fee Act (GDUFA) marks its 10‐year anniversary, quantitative methods and modeling (QMM) innovations that are driving generic drug development are thriving. This mini‐themed issue in *CPT: Pharmacometrics and Systems Pharmacology* showcases recent modeling and simulation examples to develop and assess complex generic drug products, with scientific achievements that are germane to new drug development.

The GDUFA was enacted by Congress in 2012 as a partnership between the generic drug industry and the FDA to bring cost‐saving medicines to patients faster. Under the GDUFA, the FDA assesses generic industry user fees to improve diverse aspects of regulatory review and approval of generic drug applications. Across two re‐authorizations, the GDUFA has enabled expanded research and innovation for complex generic drugs. These products are the generic version of a drug containing complex active ingredients, formulations, routes of delivery, dosage forms, and/or drug‐device combinations and for which early interactions with the FDA may be beneficial to address scientific challenges to avoid delays in approval.^2^ Due to their complexity, it is more difficult, and often prohibitive, to develop complex generics using traditional bioequivalence (BE) methods. To tackle these challenges, the Office of Generic Drugs (OGD) developed the GDUFA Science and Research Program, which has established intramural (within the FDA) and extramural (generic drug industry, academia, and the FDA) collaborations to promote innovation and application of methodologies and risk‐assessment frameworks that address scientific and regulatory issues with bringing complex generic drugs to market faster.^3^

Central to the success of the initiative is the burgeoning integration and adoption of model‐integrated evidence (MIE) by generic drug developers and the FDA. MIE integrates evidence from empirical studies with computational models, extending the continuum of knowledge acquired through model‐informed drug development (MIDD) for a new drug application (NDA) with emerging post‐NDA knowledge.^4^, ^5^, ^6^ Mechanistic modeling approaches like physiologically‐based pharmacokinetic (PBPK) and computational fluid dynamics models are extensively applied to assess substitutability. They are used by generics developers to address BE issues within abbreviated new drug applications (ANDAs) and by OGD scientists to review ANDAs and make regulatory decisions.^7^ For complex generics, MIE can use virtual BE simulations that in combination with in vitro BE testing can support alternative approaches to conventional in vivo BE studies.^4^, ^5^, ^6^

The FDA has pioneered advancements in QMM for complex generics to offer developers MIE‐driven frameworks to establish BE. The widening impact of QMM and MIE to demonstrate BE and characterize drug delivery for complex generics is spotlighted extensively in this mini‐themed issue. In 2021, workshops led by the FDA and co‐sponsored by the Center for Research on Complex Generics (CRCG) showcased advanced applications of mechanistic modeling and simulation platforms for determining BE and their utility in addressing complex generic drug development challenges. An overview of the learnings from the locally acting and oral generic drug product workshops are presented by Babiskin et al.^7^ in a mini‐review. Product category‐specific learnings are presented by Walenga et al.^8^ for oral inhalation, Tsakalozou et al.^9^ for dermal, Tan et al.^10^ for ophthalmic, nasal, injectable, and implantable, and Gong et al.^11^ for long‐acting injectable. Oral generic products, which represent a large proportion of generics, but are not routinely labeled as complex, have been identified as having lingering knowledge gaps in PBPK model development, use, and validation for absorption models and food effect assessment^12^ and as an alternative BE approach and tool for risk assessment and biowaiver.^13^ Lastly, and distinctive from the FDA workshop reports, are two model‐based BE (MBBE) applications: an intricate and comprehensive application of PBPK modeling to ivermectin to identify factors that affect plasma exposure that may be relevant to the design of complex formulations for mass‐drug administration,^14^ and an evaluation of the model‐based two one‐sided test for a single sample PK BE study with a performance comparison to noncompartmental analysis.^15^

Under the GDUFA III (2023 to 2027), utilizing QMM for complex generics to establish BE in place of clinical end point BE studies has the potential to lower development costs and accelerate access to generic drugs. A recent rise in MIE alternative BE approaches in pre‐ANDA and ANDA interactions between the generic drug industry and the FDA is signaling an industry shift from MIE development to implementation; Yoon et al.^5^ provided a forward‐looking perspective on how QMM will be pivotal to drive innovation for complex generics during this transition. The rollout of a model master file (MMF) by the OGD as a framework to communicate model practices between developers (model and product) and regulators has extraordinary potential to standardize MIE approaches and streamline regulatory review globally; consensus with the European Medicines Agency (EMA) may serve as a starting point. As Manolis et al.^16^ explained in their perspective from European regulators, whereas the EMA has broad acceptance of MIDD for NDAs, experience in assessing mechanistic modeling approaches for demonstrating BE is limited. The EMA retains an open view to build expertise in MBBE, offering ideas for platform validations with high regulatory impact applications.

As MIDD and MBBE become central tenants to innovator and generic product development, there are opportunities to hybridize learnings to advance QMM in both arenas. The MMF may find value across innovator drug development to accelerate new drug approvals as part of the MIE continuum. Curation of standards for data generation, dataset and input parameter assessment, modeling analysis and validation, and reporting to ensure consistency and harmonization across global development and regulatory decision making are pertinent to both new and generic drug development so that QMM approaches are not underutilized. The International Conference on Harmonization (ICH) M15 Concept Paper for new drug approvals provides a foundation to expand these best practices to both innovator and generic products.^17^ Finally, as applications grow for emerging practices like using real‐world evidence from postmarketing data to compare approved generic and innovator products, there may be renewed opportunities to draw parallels and synergize.
